# Pennsylvania State Dental Society

**Published:** 1884-10

**Authors:** W. H. Trueman


### PENNSYLVANIA STATE DENTAL SOCIETY.

SIXTEENTH ANNUAL MEETING, HELD AT WILKESBARRE, JULY 29, 30 AND 31, 1884.

REPORTED FOR THE INDEPENDENT PRACTITIONER BY W. H. TRUEMAN, D. D. S.

The sixteenth annual meeting of the Pennsylvania State Dental Society convened at the Wyoming Valley Hotel, Wilkesbarre, at half-past ten o'clock, Tuesday, July 29, 1884, the President, Dr. S. H. Guilford, of Philadelphia, in the chair.

Dr. C. S. Beck, of Wilkesbarre, welcomed the Society in an eloquent address, which was very warmly received.

The Committee on Enforcement of Dental Law reported that only one case had been brought to their notice ; owing to its surroundings it had been allowed to go by default. The good of the law should not be measured by the number of prosecutions brought under it. They had been few. They had ample evidence that many young men took a thorough college course who would not have done so if the law was not in existence, and had no doubt but that those men were better dentists, and were serving the communities in which they lived far better than if they had not done so. This is what the law was designed to do. They had no doubt but that in time the necessity of a proper qualification before beginning practice will be generally recognized by the public, both for dentists and physicians.

Dr. J. C. Green, of West Chester, read the report of the State Examining Board. Three gentlemen had presented themselves for examination, and had received certificates of qualification. The board was pleased to report that the candidates this year were far better prepared for the examination than any who had previously appeared before them.

Announcement was made of the death of Dr. D. T. Way, of Bedford, and Dr. T. L. Buckingham, of Philadelphia.

After the transaction of business of interest to the Society only, and which will be omitted from this report, the meeting adjourned to meet at half-past two o'clock in the afternoon.

### AFTERNOON SESSION.

The afternoon session was opened at the appointed time.

The President read his annual address, in which he reviewed the past history of the Society, and congratulated the members upon the advances that have been made through its benignant influence.

*Dr. C. N. Pierce*, of Philadelphia—Read a paper upon "Calcification and Decalcification of the Teeth."

He referred to a paper read by him at the meeting held in July, 1877, upon "The Origin and Progressive Development of Tooth-germs."

In the present paper he continued the history of the tooth, treating of the period of calcification of the teeth, and the absorption or decalcification of the roots of the deciduous teeth. In the careful examination of such animal tissues as are impregnated with lime-salts, it is evident that they, like vegetable structures, have periods of growth and of rest; and that while these conditions are normal, they are greatly modified by what we term the "function of nutrition." In studying the progressive solidification of tissues we can, with a degree of certainty, mark the beginning and the end only; the intermediate lines merely approximate, yet they are near enough to exactness to give an idea of the condition of the average tooth at a certain age to serve as an important guide in the performance of many necessary dental operations. (The doctor here referred to drawings, showing at a glance the supposed progress of calcification or decalcification of the teeth, from their first appearance in the embryo until the denture is complete.)

He briefly reviewed the development of the teeth as shown by the investigations of Dr. J. L. Williams, and especially called attention to the condition of the teeth at about the close of the third month after birth. Then the infant enters into the critical period of its life, and from a glance at the twenty deciduous teeth, it is fair to assume that this condition has not a little to do with the various abnormal systemic lesions or disturbances to which the child is liable at this age. In close proximity to the sharp and irregular edges of the calcifying extremity of each partial tooth-crown lies the vascular papilla—the primitive tooth-pulp—and any want of correspondence between the absorption of the over-laying gum at the coronal extremity, and the deposition of solid matter at the calcifying or papillary extremity, must produce by this retarding influence an irritation limited in its extent by the number of teeth advancing, and the duration of the cause. When the irritation becomes pathological it seems hardly necessary to remind dentists of the great advantage to be gained from the free use of the lance.

He then referred to the importance of knowing how far calcification had progressed when the deciduous teeth need attention early, in cases where the pulp is exposed, or nearly so. Pulps are sometimes exposed while yet the root is not completed in its growth. The impropriety of resorting to the ordinary method of pulp devitalization is, under such circumstances, very apparent.

He stated that the deciduous teeth were much less frequently subject to malformations and defects arising from deficiency in the quantity and quality of enamel and dentine than those of the permanent set, and explained that this was due to the crowns of these teeth being largely provided for in embryonic life, so that the fœtus generally escapes the consequences of imperfect nutrition, which is so common after birth. The permanent teeth, during the periods of calcification, are constantly subjected to the influence of morbid systemic conditions; should these occur while the crowns of any of the teeth are undergoing this process, markings or defects, located at the point of calcification, and limited in extent, or modified by the severity and duration of the lesion, will usually result. The fact that the third molars are developed during the period of childhood and youth, and while the system is liable to frequent conditions which impair nutrition, is probably a potent reason for their lack of usefulness and durability.

The decalcification or absorption of the roots of the deciduous teeth is a somewhat obscure physiological process, and we have found it extremely difficult to do more than approximate the time at which it is carried on. The average period at which it commences will be sufficient to indicate the time when much care will be necessary in the application of the arsenical paste for the devitalization of the pulp, and the subsequent treatment of the pulp-chamber and root-canals. We can only judge how far the process has gone on by the general condition of the mouth ; it varies so widely in different families that it is impossible to tabulate its progress with any degree of accuracy. We have spoken of this absorptive process as being physiological, and somewhat obscure. It certainly is both, and in contradistinction to the evolution of the tooth may be termed its dissolution. What induces this process has never been satisfactorily explained. That the organ has served its purpose, and that the nourishment which had previously been appropriated by it is diverted or relegated to its successor, is probably the most plausible explanation we can give of this interesting physiological process.

In recording the periods of calcification of the deciduous and permanent teeth, it should be noted that in many instances a want of correspondence between their calcification and eruption exists. By premature removal of the gum the crown is frequently exposed while yet there is no root-calcification, as instanced in deciduous incisors erupted at birth, their crowns only being calcified, which is the normal condition of these teeth at that age. Again, not infrequently the persistence of the deciduous cuspids and molars, as well as of the indurated gum over an advancing permanent molar, causes delay in the eruption of the permanent teeth until after the calcification of their roots is completed. These instances illustrate that in one case eruption takes place without the development of the root, and in the other we have complete development of both crown and root without eruption.

In closing his remarks he referred to the researches of Dr. G. V. Black, of Jacksonville, Ill., upon the same subject, stating that his conclusions closely corresponded with his own.

In the discussion which followed the reading of the paper Dr. E. T. Darby called attention to the importance of lancing the gums early, before the irritation had so far advanced as to seriously affect the child's health. If done too soon there was but little injury, while if postponed too long the consequences might be very serious, and the operation prove of but little benefit.

*Dr. Gerhart*, of Lewisburg—Asked what should be done in a case where the deciduous tooth is in place, in fair condition, and no sign of the permanent tooth, the patient having passed the age when the change should have taken place. Some cases in which he had not removed the deciduous tooth had resulted in serious irregularity, and in other cases where he had done so, the permanent tooth had failed to appear. He knew a gentleman over forty-five years of age with more deciduous teeth than permanent.

Several expressed the opinion that it was best not to remove a deciduous tooth, in fair condition, until there were indications of the permanent tooth. It might be that the germ of the permanent tooth was absent, and in that case the deciduous tooth was better than none, as long as it would remain. On the other hand, it was suggested that if the deciduous tooth was removed and the germ was there, the absence of the baby tooth would probably stimulate its development; if the germ was not there the teeth would close up, and an unsightly space be avoided.

Subject passed.

(TO BE CONTINUED.)

